# p53 as an intervention target for cancer and aging

**DOI:** 10.3402/pba.v3i0.22702

**Published:** 2013-10-08

**Authors:** Paul Hasty, Barbara A. Christy

**Affiliations:** 1 Department of Molecular Medicine, Institute of Biotechnology, University of Texas Health Science Center at San Antonio, San Antonio, TX, USA; 2 Cancer Therapy & Research Center, University of Texas Health Science Center at San Antonio, San Antonio, TX, USA; 3 Barshop Institute for Longevity and Aging Studies, University of Texas Health Science Center at San Antonio, San Antonio, TX, USA

**Keywords:** DNA damage, cell growth, cellular senescence, apoptosis, anaerobic glycolysis

## Abstract

p53 is well known for suppressing tumors but could also affect other aging processes not associated with tumor suppression. As a transcription factor, p53 responds to a variety of stresses to either induce apoptosis (cell death) or cell cycle arrest (cell preservation) to suppress tumor development. Yet, the effect p53 has on the non-cancer aspects of aging is complicated and not well understood. On one side, p53 could induce cellular senescence or apoptosis to suppress cancer but as an unintended consequence enhance the aging process especially if these responses diminish stem and progenitor cell populations. But on the flip side, p53 could reduce growth and growth-related stress to enable cell survival and ultimately delay the aging process. A better understanding of diverse functions of p53 is essential to elucidate its influences on the aging process and the possibility of targeting p53 or p53 transcriptional targets to treat cancer and ameliorate general aging.

p53 is a transcription factor whose ability to suppress tumorigenesis in mammals has been extensively studied (1–3), but emerging evidence suggests that p53 also influences the aging process and longevity independent of its role as a tumor suppressor. However, just how p53 influences general aging is not well understood. p53 regulates the transcription of a large number of genes with a myriad of anti-oncogenic functions that include cell cycle arrest (p21, GADD45, 14-3-3σ, RPRM), apoptosis (Scotin, Killer, FAS, BBC3, PERP, 53BP1, BAX, LRDD, PMAIP1), suppression of aerobic glycolysis (GLUT1, TIGAR, hexokinase, phosphoglycerate mutase), facilitation of oxidative phosphorylation (OXPHOS) (SCO2, AIF), cell growth (PTEN, AMPK beta, TSC2, IGF-BP3) (4), and protein translation (sestrins) (5). p53 also has transcription-independent activities that include regulating microRNA processing (6), DNA repair (7), mitochondrial protein survival (8), and ribosome biogenesis (9,10). Thus, p53 is critical for maintaining genome integrity (ploidy and structure) and regulating both cell growth (mass) and cell proliferation (number) during times of stress. These activities are central for tumor suppression (11) but could also influence aging and longevity independent of oncogenesis. Evidence that p53 influences lifespan through a non-cancer-related mechanism also comes from knockdown of the *Caenorhabditis* *elegans* p53 gene (Cep-1) because it increases lifespan (12). Furthermore, human epidemiological studies show a polymorphism resulting in an amino acid change in the p53 protein at codon 72 (Arg–Pro) decreased apoptotic potential (13) and led to increased cancer risk. However, this change also increased survival supporting the possibility that p53-mediated apoptosis prevents cancer but also contributes to aging (13–15). As a result, therapeutic strategies are being designed and tested to enhance p53 function for those tumors that maintain functional p53 and these same therapeutics or interventions could be useful to ameliorate or delay aging (Fig. 1).

**Fig. 1:**
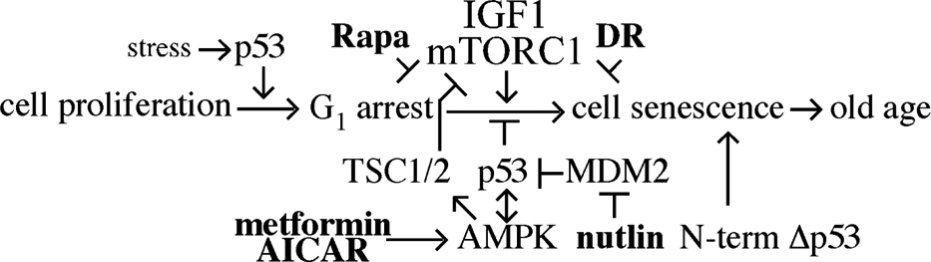
The tumor suppressor p53 integrates multiple stressor signals with DNA damage and growth responses. Multiple interventions that influence p53 function are available (shown in bold) that have the potential to suppress cancer and potentially organismal aging. Yet, because of the complicated integration of p53 with cell stressors and pro-growth pathways direct p53 intervention could have a multifactorial impact that could enhance cellular senescence and aging.

## Mouse models for p53 and aging

There are numerous mouse models with altered p53 activity that demonstrate the effect that p53 has on aging and longevity in addition to its role in tumor suppression. These models range from mice homo- or heterozygous for a simple null mutation in p53 to mice containing point mutations that disrupt some biochemical functions of p53 but leave others intact. Mice also exist with N-terminal truncations that mimic some of the naturally occurring isoforms found in human tumors and normal cells. A review of these models demonstrates the complexity of p53 function at the organismal level.

The first p53 mouse models were simple null mutations. p53 null mice develop normally but a subset of the mice exhibit an overgrowth of neural tissue in the region of the mid-brain to cause exencephaly because of defective apoptosis (16). However, most *p53* ^*−/−*^ mice survive to become apparently normal adults. Yet, the majority of these mice develop cancer at about 6 months of age (usually lymphomas and sarcomas); heterozygous *p53* ^*+/−*^ mice also succumb to cancer before control mice but at a later age than *p53* ^*−/−*^ mice (17). Tumors from *p53* ^*+/−*^ mice often show loss of heterozygosity, but mutation of one p53 copy can cause a haploinsufficiency (18). The genetic background and exposure to chemical carcinogens influence cancer onset and spectrum (19–22). Thus, p53 null mouse models expose the role of p53 and its importance during development and for tumor suppression. Because mice deficient in p53 succumb to certain types of cancer at an early age, these models are not very useful to study the role of p53 in aging or even in the study of cancers that develop in old age (which includes most tumor types in humans). In addition, because p53 function appears to be cell-type specific and affects different tumor types differently (23), germline null mutations might not be the best approach. The use of conditional knockouts has shown that p53 loss can promote tumorigenesis in epithelial cells, at least in combination with other genetic changes causing stress (24). Thus, the study of conditional and tissue-specific knockouts in combination with factors important in aging will likely provide more information on the role of p53 in the aging process.

In contrast to p53 deletion, p53 overexpression from a BAC reduced cancer levels without affecting aging (25). In addition, overexpression of Arf and p53 together further lowered cancer levels to extend lifespan (26). Similarly, reducing MDM2 (a negative p53 regulator) increased p53 levels to lower cancer incidence without affecting aging (27). Thus, increased wild-type p53 levels appear to reduce cancer incidence without influencing aging. Theoretically, increased p53 dose alone will not automatically lead to increased p53 activity in normal cells, because p53 is normally under strict control and becomes stabilized and active only in times of cellular stress. Perhaps, increased p53 levels will be toxic only when the level exceeds the capacity of those proteins that serve to keep p53 activity under control.

However, increased p53 levels can be toxic and cause death or actually accelerate aging in certain settings. For example, p53 dose influenced the replicative lifespan for fibroblasts in tissue culture (28,29). p53 is also essential for causing the characteristic premature replicative senescence in fibroblasts defective for a variety of DNA repair genes (30,31). In addition, p53 causes embryonic lethality in *Mdm2*- or *Mdm4*-mutant mice (32–34). Although the p53 in these mice is normal, p53 is not regulated properly because of the mutations in these two negative regulators (but lethality can be rescued by deletion of p53). p53 dose also influences the early aging phenotype observed in mice deficient in the Brca1 breast cancer susceptibility gene. Deleting one copy of p53 rescued *brca1* ^*−/−*^ mice from early embryonic death, only to result in early aging (35,36). In addition, mice deleted for the REG (11S regulatory particles, 28-kDa proteasome activator) γ accumulate casein kinase (CK) 1δ and p53 and exhibit characteristics of early aging (37). Reduced CK1δ activity inhibits MDM2-mediated degradation leading to increased p53 levels and p53 haploinsufficiency ameliorated early aging, while increased MDM2 attenuated cellular senescence for fibroblasts. These results suggest CK1δ-Mdm2-p53 regulation plays an important role in cellular aging. However, REG has other targets besides p53 and CK1 that could contribute to the aging phenotype. In addition, skin-specific MDM2 deletion caused p53-mediated senescence in epidermal stem cells and early aging in the skin (38). Therefore, the levels and activity of p53 are clearly important both in embryonic development and aging in cells and in mice. It appears that normal p53 causes accelerated aging or senescence mainly when it is not regulated properly or when some kind of stress is present, including DNA damage response defects, Brca1 defect, or proteasome dysfunction.

There are multiple isoforms for p53 and the related p53 family members (p63 and p73); they are produced by different promoter usage, alternative splicing, and alternative translation initiation sites (39,40). These p53-related isoforms could influence cancer differently, but their functional differences are not well understood (41). In addition to having unique properties, p53 isoforms could interact with full-length p53 and modulate its activity. Differences in isoform ratios could influence development, cell proliferation, stress responses, and cancer and aging. For example, expression of an N-terminally truncated p53 called p44 generated by nonsense mutations in the p53 N- terminus caused urinary bladder tumors (42). Gene expression profiles of embryonic stem (ES) cells carrying p44 in a p53 null background are divergent from those of p53 null ES cells, suggesting p44 has transcriptional properties independent of full-length p53 (42). In addition, a particular p53 isoform appears to contribute to the ATR-intra-S phase checkpoint that selectively transactivates *p21* and *14-3-3*σ but not the *Mdm2*, *Bax*, and *Pig3* promoter (43). Such differences in activity for different p53 isoforms could influence cancer. Consistent with this idea, variable expression of p53 isoforms has been observed in breast tumors compared to normal breast tissue (39,44). Thus, different p53 isoforms might have unique activities that influence oncogenesis.

Isoforms of p53 with N-terminal truncations could conceivably enhance aging since their overexpression reduced cancer but accelerated aging in mice (45,46). These mice exhibited shortened lifespans and premature aging characteristics similar to those described for the DNA-repair-deficient mouse models (30). In addition, overexpression of N-terminal truncated p53 isoforms reduced tissue function and regeneration, suggesting a defect in stem and progenitor cells (47). In support of this notion, deletion of one p53 copy increased the number of proliferating hematopoietic stem and progenitor cells in old mice, whereas mice that express an N-terminal truncated p53 with increased activity did not show this increase (48). Defects in mammary gland ductal morphogenesis were also observed (49). To realize the premature aging phenotype in mice expressing truncated p53, full-length wild-type p53 was also required, suggesting that the truncated isoforms associated with full-length p53 as a tetramer. In support of this possibility, one of the N-terminal truncated p53 isoforms was observed to interact with full-length p53 to increase stability and nuclear localization even in the absence of stress (50). Another isoform stabilized p53 in the presence of Mdm2 (51). Thus, overexpression of a particular p53 isoform likely influences the other isoforms and the overall p53 function.

Mutations in p53 are found in half of all human cancers, and other parts of the p53 pathway are altered in many others. These mutations confer a selective advantage on the tumor cells, allowing them to evade cell cycle checkpoints, avoid apoptosis and senescence, and proliferate under conditions where normal cells cannot. Most tumor-associated p53 mutations are within the DNA-binding domain. These affect the ability of p53 to bind to target genes to varying degrees, and hence may alter the overall protein conformation. Most can still tetramerize with wild-type p53 and exert a dominant negative effect. Many engineered mutations in p53 that affect its regulation and function have been tested in cell culture and in mice. Although studies of function must generally start by utilizing tissue culture systems, the use of mouse models is important to interrogate the complex processes of tumorigenesis.

It would be difficult to identify analogous mutations that affect aging, because they would presumably not be selected in the same manner. However, a polymorphism recently identified in human populations suggests that subtle changes to the p53 protein can also affect longevity and aging. A simple substitution of a proline for an arginine at codon 72 hinders the ability of p53 to induce apoptosis and leads to an increased cancer risk. However, this polymorphism was enriched in the older population and was associated with longevity, even though some had died from cancer (13–15). We predict that many more such mutations and polymorphisms will be found that affect the aging process, independent of cancer.

## Activation of p53

A number of studies have shown some benefit in targeting endogenous wild-type p53 or mutant p53 in order to enhance its function and slow tumor growth. The most promising approach involves drugs able to reactivate unfolded or mutant p53 (as well as the related p63 and p73 proteins in some cases). Because of its diverse activities and profound biological outcomes, p53 is tightly regulated by multiple mechanisms that control activity, stability, and localization (Fig. 1). As a consequence, enhanced p53 regulation can lead to cancer. Thus, targeting p53 regulation could effectively reactivate p53 in those cancers.

MDM2 (murine double minute 2) and MDM4 (a.k.a. MDMX) regulate p53 and their overexpression effectively negates functional p53 to enhance cancer risk. MDM2 is a ubiquitin ligase that leads to degradation of p53. When MDM2 levels are high, p53 becomes polyubiquitinated and targeted for degradation by the proteasome. MDM2 also directly represses p53 activity. The MDM2 gene is also a target for p53, setting up a negative feedback loop. When p53 is activated, MDM2 levels increase, which then turns down p53. MDM4 is not a ubiquitin ligase but enhances p53 ubiquitination in a heterocomplex with MDM2. MDM4 also regulates p53-mediated transcription. MDM2 and MDM4 are not redundant. Deletion of either protein causes embryonic lethality in mice, and p53 deletion rescues either mutation (32–34). Apoptosis and cell cycle arrest caused MDM2- and MDM4-mutant embryos to die, respectively (52). Heterodimerization of MDM2 and MDM4 is important for regulating p53 during embryogenesis but not in the adult mouse (53). Furthermore, MDM2 appears to regulate p53 stability whereas MDM4 regulates p53 activity (54). There are also tissue-specific differences in the utility of MDM2 and MDM4 in p53 regulation (55). Thus, MDM2 and MDM4 function both together and independently to regulate p53; making each a potential target for anti-cancer therapy.

At least half of all tumors are mutated for p53, whereas many other tumors with wild-type p53 have an effectively dysfunctional p53 pathway because of overexpression of MDM2. Therefore, small molecules that interfere with MDM2/p53 binding could effectively restore p53 activity without causing genotoxic stress. This strategy would be particularly effective for pediatric cancer since those tumors usually contain wild-type p53 (56). The *cis*-imidazoline analogue Nutlin-3 disrupts the p53–MDM2 interaction to enhance p53 function and promises to be an alternative to chemotherapy. Nutlin-3 also shows a synergistic effect in combination with certain therapeutics, such as TRAIL or bortozemib (57), or in combination with ionizing radiation (58). Even though Nutlin-3 and other compounds inhibit MDM2/p53 binding, their therapeutic index is not known (59).

MDM4 is also a potential target for intervention to enhance p53 function since MDM2 and MDM4 have a complementary mode of action (60). Importantly, restoration of p53 function in the absence of MDM4 enhanced lifespan in a mouse tumor model (61) and temporary restoration of p53 in the absence of MDM4 is non-lethal. Furthermore, a mouse p53 mutant deleted for the proline-rich domain (p53^Δp^) showed enhanced p53^Δp^-mediated suppression of oncogene-induced tumors after MDM4 loss, supporting MDM4 as an anti-cancer target (54). So far, compounds that disrupt the MDM4–p53 interaction have not been found. Thus, systemic MDM4 inhibition is a sound approach for restoring p53 function in tumors with functional p53 and could potentially be less toxic than MDM2 inhibition.

Besides the small molecule Nutlin, several compounds appear to be able to activate p53 by altering its conformation (62). The small molecule CP-31398 restores p53 activity in tumor cells containing at least some p53 mutations, apparently by blocking ubiquitination and thus degradation without interfering with MDM2 interaction. CP-31398 may also be able to activate the p53 family members p73 and p63, contributing to anti-tumor effects. More recently, the chemical PRIMA-1 (p53 reactivation and induction of massive apoptosis) has shown promise in mediating p53-dependent apoptosis in tumor cells expressing mutant p53 (63). The PRIMA-1 derivative APR-246 is able to activate unfolded wild-type p53 as well as mutant p53; suggesting that it may work in tumors with or without mutated p53. APR-246 has recently undergone testing in human clinical trials and appears safe and well tolerated (64). Initial tests indicate that activation of p53 was seen in human patients with hematologic malignancies and prostate tumors (64), but subsequent testing on its anti-tumor efficacy will be needed. It will also be necessary to identify any effects of these drugs on the aging process if long-term chronic treatment of humans is proposed. In any event, activating the tumor suppressive effects of p53 appears to be a promising treatment strategy that may decrease the necessity of using highly toxic chemotherapeutic drugs in the future (65).

## Posttranscriptional regulation of p53: phosphorylation and acetylation

In addition to ubiquitination, other posttranslational p53 modifications include phosphorylation and acetylation, neddylation, sumoylation, and methylation, all of which are essential for modulating p53 activity. p53 is a highly regulated protein that inhibits cell proliferation and induces apoptosis or senescence in response to a variety of cellular stresses, including DNA damage, oxidative stress, hypoxia, and oncogenic signaling. In normal cells that are not under stress, p53 levels and activity are very low. When cells are subjected to stress, p53 protein levels increase and p53 transcriptional activities are activated. This is not an all or none (on or off) situation; p53 activity is likely to be regulated in a graded manner, and different activities of p53 are undoubtedly regulated differentially by different modifications. These modifications can profoundly affect the p53 activity in tumor suppression, and are likely to affect the effects of p53 on aging as well.

The p53 protein has multiple Ser/Thr residues that serve as phosphorylation sites for a number of protein kinases. The phosphorylation state of many of these sites changes upon stress signaling. Many important regulatory sites are concentrated in the transactivation domain at the N-terminus, with some also present in the C-terminal regulatory domain. Some sites can be phosphorylated by multiple kinases and some kinases can phosphorylate more than one site. Two important sites are Ser 15 (Ser 18 in mouse p53) and Ser 20 (Ser 23 in mouse). These sites become phosphorylated by ATM and other kinases in response to DNA damage. When these serines are phosphorylated, the interaction with the negative regulator MDM2 is weakened, stabilizing p53 and allowing it to interact with coactivators and activate transcription. Mutant mice with a Ser to Ala mutation at these sites (cannot be phosphorylated) show increased p53 stability and transactivation. However, the phenotype is tissue specific and not as severe as would be predicted from the effects in tissue culture experiments, suggesting that phosphorylation is not the only means of controlling p53. Interestingly, mice generated by a knock-in of the Ser 18 (Ser to Ala) mutation show accelerated aging, suggesting that active p53 in response to DNA damage protects against aging (66). A knock-in mouse model that mimics constitutive phosphorylation and activation of p53 has also been generated and shows a striking aging phenotype (67). These mice show large amounts of stem cell apoptosis in several organs, preventing proper tissue renewal. Depletion of the pro-apoptotic p53 target PUMA led to stem cell rescue in these mice, blunting the accelerated aging phenotype. This suggests that controlling apoptosis and the proliferative capacity of stem cells may be behind the effect of p53 on aging.

Acetylation is a major mechanism for regulating p53 activity. Multiple lysine residues in p53 are acetylated; many of these same lysines are also targets for ubiquitination by MDM2. Therefore, acetylation of these lysine residues prevents their ubiquitination, stabilizing p53. In addition, acetylation can inhibit the interaction of MDM2 with p53. Finally, acetylation recruits cofactors allowing p53 to activate its transcriptional targets. Acetylases that are responsible for modification of p53 include p300, CBP, PCAF, TIP60, and hMOF (68). In tissue culture experiments, acetylation stabilizes p53 and enhances its activity, because it competes with ubiquitination. However, in mouse knock-in experiments, mutants with multiple lysines in the C-terminal regulatory region changed to Arginine (cannot be acetylated) do not show large defects in apoptosis, cell cycle arrest, or tumor suppression. Two acetylation sites in the DNA-binding domain (where most tumor-associated mutations occur) may be more important. K120 acetylation by TIP60 appears to be necessary for activating genes encoding proteins important in apoptosis but not cell cycle arrest. Thus, modification of this site may be important for choice of target genes in response to particular stress signals. In contrast, K164 acetylation appears to be necessary for activation of most p53 target genes. If both K120 and K164 along with the six C-terminal lysines are mutated so that they cannot be acetylated, p53 is rendered inactive for the ability to cause cell cycle arrest or apoptosis (69). Deacetylase enzymes also work on p53 to counteract acetylase activity. In particular, SIRT1 acts on K382 and negatively regulates p53 action on pro-apoptotic genes. The compound resveratrol, found in grapes and red wine, activates SIRT1 and is a potential anti-aging target; thus it represents a promising means to control p53 activity during aging. However, SIRT1 can either promote or suppress tumors, depending on context, so care must be taken to avoid detrimental effects of SIRT1 augmentation.

Recent work showed that manipulation of p53 acetylation in mice could separate the classic p53 functions of cell cycle arrest, induction of apoptosis, and senescence from the anti-cancer activity of p53 (70). In this study, mice were generated by knocking-in a p53 allele with three acetylation sites in the DNA-binding domain mutated to arginines to prevent acetylation. This mutant (3KR) still binds to DNA, but does not induce p21 or PUMA although it still induces MDM2 transcription. In mice, the mutant p53 can activate transcription of some genes, but it is defective in p53-dependent cell cycle arrest, p53-mediated apoptosis in thymocytes, and p53-mediated senescence in mouse embryonic fibroblasts (MEFs). Surprisingly, the characteristic early tumors seen in p53 mutant mice were abrogated in these knock-in mice. Thus, at least for the early tumors caused by germline p53 loss, the ability of p53 to mediate cell cycle arrest, apoptosis, and senescence is not needed (70). It will be interesting to see how the aging process is affected in these mutant mice.

There are other ways p53 can be modified. p53 can be methylated on lysines and arginines by several different methylase enzymes, which can either activate or repress its activity depending on which site is methylated and the number of methyl groups added (69). p53 can also be modified on lysine residues by two other ubiquitin-like molecules, SUMO (small ubiquitin-like modifier) and NEDD8 (Neural precursor cell Expressed Developmentally Downregulated protein 8) (69). So far, the effects of these modifications are less well studied than ubiquitination, although they can modulate p53 activity experimentally. It remains to be seen what the effects of these modifications on aging are.

It is clear that p53 protein activity can be modulated by several types of posttranslational modification, and that the overall activity of a given p53 protein molecule will be determined in a complex manner by the balance between different amounts and combinations of modifications at any given time. Some of the modifications are likely to affect other modifications, particularly those that target the same p53 amino acid residues. Affecting p53 activity by altering amounts, timing, and specificity of protein modification represents a promising avenue to affect the aging process.

## p53 and ribosome biogenesis

Ribosome biogenesis refers to the production and processing of ribosomal RNA. It is a complex process that occurs in the nucleolus of the cell. RNA polymerase I (Pol I) transcribes tandem arrays of ribosomal DNA (rDNA) (about 400 repeats in humans) in the nucleolar organizer region (NOR) to delineate a nucleolus (71). This process consumes about 80% of the energy in proliferating cells. Specialized ribosomes that selectively translate mRNAs are hypothesized to occur because of differential expression and posttranslational modifications of ribosomal proteins along with rRNA diversity and ribosome-associated proteins making this process potentially very complex (72). Defective ribosome biogenesis can alter protein expression that causes ribosomopathies (73). Cancer cells upregulate ribosome biogenesis and mutations in ribosomal protein genes suggest upregulation induces oncogenesis (74).

Interestingly, ribosome biogenesis serves as a sensor of cellular stress and defects in ribosome biogenesis activate p53 (75). Furthermore, DNA damage has to occur within the nucleolus in order to induce a p53-mediated DNA damage response (76). Yet, defective ribosomal biogenesis without DNA damage also stabilizes p53 (74). Ribosomal biogenesis stress causes the ribosomal proteins (rp) L11, L5 and L23 to interact with MDM2, thereby stabilizing p53 (77–79). Ribosomal stress induces apoptosis that depends on both rpL11 and p53 in mouse pluripotent stem cells (80). In addition, the oncoprotein c-Myc stabilizes p53 through rpL11-mediated HDM2 (the human ortholog to MDM2) inhibition, and the ASK1/p38 kinase activates p53 through phosphorylation on serine 15 and 33 (81). However, rp5, rpL11, and rp23 are needed to stabilize p53, not serine 15 phosphorylation (82,83). Ribosome biogenesis is connected to cell cycle regulation because p53 monitors 18S and 28S rRNA synthesis through rpL11 (82,83). Thus, ribosomal stress (with or without DNA damage) causes a p53 response.

Inhibitors of ribosome biogenesis (genotoxic or non-genotoxic agents) that induce a p53 response could potentially function as potential anti-cancer agents (74). DNA damaging agents disrupt nucleolar morphology suggesting that they inhibit ribosome biogenesis. These agents disrupt rRNA transcription by different methods. Crosslinking agents and inhibitors of dihydrofolate reductase or type 1 topoisomerase suppress RNAPI transcription, whereas the intercalating agent actinomycin D inhibits rRNA elongation. Non-genotoxic agents that inhibit any of the three RNA polymerases will also inhibit ribosome biogenesis, including α-amanitin which specifically inhibits RNAPII. There are also cyclin-dependent kinase (CDK) inhibitors such as roscovitine and 5,6-dichloro-1-β-d-ribofuranosylbenzimidazole (DRB) that interfere with 47S rRNA processing and the antimetabolite 5-fluorouracil (5-FU) that likely reduces rRNA pseudouridylation and the formation of functional snRNA; thus, inhibiting RNA metabolism as opposed to DNA metabolism. In addition, inhibiting endoribonuclease activity of NPM1 blocked 32S rRNA processing into 28S rRNA to induce cell death. Finally, interfering with pro-growth pathways like mechanistic target of rapamycin (mTOR) or activation of c-Myc oncoprotein would decrease rRNA transcription and processing. Inhibiting the AKT protein kinase also decreased RNAPI transcription. Thus, a variety of targets are available to inhibit ribosome biogenesis to induce a p53 response (74). However, the effects of inhibition of ribosome biogenesis as a means to induce p53 activity remain to be determined.

## p53 and mTORC1

Cellular senescence is a permanent arrest in the ability of a cell to proliferate and has been proposed to contribute to organismal aging; yet, the precise mechanism that drives cells into senescence is not understood. A common belief is that overexpression of p53 causes G_1_-arrested cells to transit from quiescence (where they still retain the ability to proliferate) to senescence. However, p53 might not induce cellular senescence beyond G_1_ arrest, so its role in senescence induction is unclear (84). p53 could actually inhibit cellular senescence (gerosuppression) under some conditions (85). To support this possibility, p53 overexpression favored quiescence in cells that would otherwise undergo senescence because of p21 overexpression (86). Furthermore, the pro-growth mechanistic target of rapamycin complex 1 (mTORC1) could contribute to the induction of senescence in p21 expressing cells (geroconversion) (85). To support this possibility, mTORC1 overexpression (via TSC2 knockdown) caused quiescent cells to enter senescence after nutlin-3a-mediated growth arrest (87). The mTORC1 inhibitor, rapamycin reversed this process. Similarly, the mTORC1 negative regulator, TSC1 maintained naive T cells in a quiescent state (88). Thus, p53 in concert with mTORC1 could be important for induction of cellular senescence.

mTOR is a serine/threonine kinase in the phosphatidylinositol 3-kinase (PI3K)-related family (89–92) and is found in a complex (mTOR complex 1; mTORC1) that is conserved across species from yeast to mammals. mTORC1 regulates cell growth (mass) and proliferation (cell division) in response to environmental signals, thereby ensuring that proliferation occurs only when sufficient nutrients are available without destructive stresses such as DNA damage. Growth factors like IGF-1 bind to receptor tyrosine kinases like IGF-1R in the cell membrane to activate PI3 kinase leading to phosphorylation of PIP3 (phosphatidylinositol-3,4,5-triphosphate), a second messenger in the cell membrane. The tumor suppressor PTEN (phosphatase and tensin homologue deleted on chromosome 10) attenuates this pathway through dephosphorylating PIP3. PIP3 activates AKT which inhibits TSC1/TSC2 (tuberous sclerosis complex), which in turn acts as a negative regulator of mTORC1 (93,94) . Activated mTORC1 upregulates mRNA translation to enhance protein synthesis. mTORC1 directly phosphorylates S6K1 (S6 kinase 1) to induce ribosome biogenesis and translation (90,91). In addition, mTORC1 phosphorylates 4E-BPs (elF4E-binding proteins) to terminate binding to elF4E and relieve the block on translation. Rapamycin specifically inhibits mTORC1 (95) by binding to a protein folding chaperone (FKBP12) required for mTORC1 activity (96). Thus, mTORC1 enables cell growth in response to mitogenic signaling in the presence of high nutrient/energy levels and low stress (84).

Rapamycin has been shown to increase lifespan for heterogeneous outbred mice (97,98) and for C57BL/6 inbred mice (99). In part, rapamycin extended lifespan through cancer suppression. But rapamycin also ameliorated non-cancer-related signs of aging in mice (99–101). It is possible that p53 complements anti-cancer and anti-aging effects of rapamycin because p53 inhibits the mTORC1 pathway through AMPK (5′-adenosine monophosphate-activated kinase) phosphorylation of the TSC1/2 complex (92,102). In addition, mTORC1 increased p53 activity in response to DNA damage (103). Thus, both ATM and mTORC1 induce p53 suggesting an integration of the DNA damage response with energy levels (84). These observations suggest that at least part of effect of rapamycin on lifespan and non-cancer-related aging is through indirect reduction the p53 stress response.

## p53 and glucose metabolism

Glucose is the major source of energy for ATP generation and glycolysis is an ancient metabolic pathway that converts glucose to pyruvate to produce ATP and NADH. Pyruvate then enters the tricarboxylic acid (TCA) cycle to generate ATP through OXPHOS. Contrary to normal cells, cancer cells primarily generate energy (ATP) through aerobic glycolysis to increase anabolism and perpetuate tumor growth, known as the Warburg effect (104). In addition, mutations that diminish the TCA cycle are oncogenic. For example, mutations in the TCA enzyme, fumarate hydratase (FH) lead to hereditary leiomyomatosis (that causes cutaneous and uterine leiomyomas) and renal cell carcinoma (HLRCC) that causes type II papillary kidney cancer (105). These cancers exhibit aerobic glycolysis and impaired OXPHOS. This means that alterations in energy production contribute to oncogenesis.

As an important aspect for tumor suppression, p53 suppresses this metabolic shift from OXPHOS to aerobic glycolysis to generate ATP (106). p53 negatively regulates glycolysis by multiple mechanisms. First p53 reduces expression of glucose transporters (107) and represses the insulin receptor promoter (108). p53 also negatively regulates phosphoglycerate mutase (PGM) by controlling protein stability (109) and by transactivating TP53-induced glycolysis and apoptosis regulator (TIGAR) (110); thus reducing glycolysis and diverting glycolytic intermediates to the pentose phosphate pathway (PPG). p53 also suppresses carbohydrate responsive element-binding protein (ChREBP), which supports aerobic glycolysis (111). In addition, p53 enables OXPHOS by upregulating aerobic respiration through its direct transcriptional target synthesis of cytochrome *c* oxidase 2 (SCO2) (112,113) and the mitochondrial apoptosis-inducing factor protein (AIF) (114,115) that are important for complex IV and complex 1, respectively. Thus, p53 regulates metabolism to suppress aerobic glycolysis and enhance OXPHOS.

Oroxylin A is a bioactive flavonoid found in the roots of *Scutellaria baicalensis* Georgi that induces apoptosis in HeLa cells (116) via p53 (117). Yet, at lower concentrations, Oroxylin A reduces aerobic glycolysis through p53 induction of TIGAR and SCO2; thereby inhibiting the Warburg effect (118). Oroxylin A induces phosphorylation of p53 serine 15 and suppressed MDM2 expression (118). Thus, upregulation of p53 can reduce aerobic glycolysis to suppress the growth of tumor cells.

p53 regulates glycolysis and OXPHOS in response to oxidative stress and energy and amino acid metabolism (119). Metabolic stress activates p53 through AMPK to regulate insulin-like growth factor (IGF-1)/AKT and the mTORC1 pathways via transcription of a variety of genes including PTEN (115), TSC2 AMPK b1, Sestrins 1 and 2 and REDD1. p53 increases the expression of sestrins 1 and 2 (which in turn activate AMPK (AMPK pThr172), and TSC2 (which inhibits mTORC1)). Thus, p53 negatively regulates pro-growth pathways as a part of a stress response.

Metformin and 5-aminoimidazole-4-carboxamide-1-β-d-ribofuranoside (AICAR) induce AMPK and as a result induce p53. Metformin also inhibits mTORC1 via AMPK (120). Metformin reduces the ill effects of diabetes mellitus type 2 (including increased cancer risk) that are potentially because of activation of the insulin- and IGF-signaling pathways. Metformin was shown to inhibit melanoma invasion (but not migration or proliferation) dependent upon activation of AMPK and p53 (121). Metformin also inhibits growth and enhances radiation response for non-small cell lung cancer through AMPK and p53 (122). Similar to metformin, AICAR induces AMPK and was found to increase p53 phosphorylation and elevate p21 to arrest endothelial cells in G_0_ or G_1_ (123). Thus, AMPK-activating agents have the potential to induce p53 and reduce mTORC1 to suppress cancer and possibly other diseases.

## p53 and caloric restriction

Restricted food intake or caloric restriction (CR) is a well-characterized intervention to extend lifespan and ameliorate physiological aging across species, including mammals (124). CR causes many changes related to lifespan extension (125,126); thus, understanding the most efficacious changes is difficult (127). CR activates sirtuins that are NAD(+)-dependent deacetylases (128,129) and p53 was proposed to connect mTORC1 and SIRT1 to influence cell survival in response to CR (130). A natural compound found in wine, resveratrol (3,5,4′-trihydroxystilbene) activates the sirtuin, SIRT1 (131) and extends the lifespan for mice on a high-calorie diet (132), suggesting that resveratrol might mimic some of the beneficial effects of CR without actually restricting calories (a benefit for human intervention). These mice showed increased insulin sensitivity, reduced IGF-I and increased AMPK (known to induce p53 and inhibit mTORC1). Thus, life extending effect of CR could be partly because of AMPK/p53 inhibition of mTORC1. Functional p53 is not necessary for lifespan extension by CR in mice, since the lifespan of *p53* ^*−/−*^ mice is increased by this intervention (133). However, the lifespan extension in these mice that are prone to early tumors may be entirely because of a delay in tumor formation or progression, rather than an effect on the aging process. CR begun later in life is still effective to delay tumorigenesis in *p53* ^*+/−*^ mice (134). It remains a possibility that the p53-related proteins (p63 and p73) could substitute for p53 in response to CR. Alternatively, important targets in CR may be located downstream from p53.

## Conclusion

p53 connects cell metabolism and DNA integrity to various cellular outcomes including cell cycle arrest, cellular senescence, and cell death. Although the role of p53 in tumor suppression has been well studied, recent evidence suggests that p53 also affects aging. There are multiple ways to target p53 as an anti-cancer therapeutic (Fig. 1). However, directly targeting p53 to suppress aging phenotypes would be difficult considering the delicate balance that is needed among arrest, senescence, and apoptosis. Animal models highlight these complexities. It is possible that p53 induces a G_1_ arrest in response to damage or cellular stress to protect the cell, but does not induce cellular senescence directly. Instead pro-growth pathways may enable an arrested cell to enter a senescent state. Recent data that show p53 allows cells to survive serine depletion highlight a protective role (135). These observations imply that specific and narrow interventions to either up or downregulate p53 activity might be suitable for cancer but not effective for general aging. Instead, broad interventions that reduce growth (rapamycin, CR, resveratrol) or mimic reduced growth (metformin, AICAR) may be the best candidates to alter p53 function in a manner that ameliorates or slows aging.
